# Severity of Anemia During Pregnancy and Adverse Maternal and Fetal Outcomes

**DOI:** 10.1001/jamanetworkopen.2021.47046

**Published:** 2022-02-03

**Authors:** Huifeng Shi, Lian Chen, Yuanyuan Wang, Mengxing Sun, Yijie Guo, Shang Ma, Xiaoli Wang, Hai Jiang, Xiaoxia Wang, Jie Lu, Lin Ge, Shu Dong, Yu Zhuang, Yangyu Zhao, Yuan Wei, Xudong Ma, Jie Qiao

**Affiliations:** 1 Department of Obstetrics and Gynecology, Peking University Third Hospital, Beijing, China; 2 National Clinical Research Centre for Obstetrics and Gynaecology, Beijing, China; 3 National Centre for Healthcare Quality Management in Obstetrics, Beijing, China; 4 School of Biological Science and Medical Engineering, Beihang University, Beijing, China; 5 Department of Maternal and Child Health, Peking University School of Public Health, Beijing, China; 6 Department of Medical Affairs, Peking University Third Hospital, Beijing, China; 7 Department of Healthcare Quality Evaluation, Bureau of Medical Administration, National Health Commission of the People’s Republic of China, Beijing, China

## Abstract

**Importance:**

Anemia is the most widespread nutritional deficiency among pregnant females in the world. Despite numerous studies on anemia, evidence is limited about the association of severity of anemia with maternal and fetal health.

**Objective:**

To investigate the association between severity of anemia during pregnancy and risk of maternal and fetal adverse outcomes.

**Design, Setting, and Participants:**

This retrospective cohort study used data from China’s Hospital Quality Monitoring System from January 1, 2016, to December 31, 2019, for pregnant females aged 15 to 49 years with birth outcomes reported at 1508 hospitals with maternity services in mainland China.

**Exposures:**

Anemia of varying severity during pregnancy was identified from daily standardized electronic inpatient discharge records using corresponding codes of the *International Statistical Classification of Diseases and Related Health Problems, Tenth Revision*. Mild anemia was defined as a hemoglobin concentration of 100 to 109 g/L (to convert g/L to g/dL, divide by 10.0); moderate anemia, as 70 to 99 g/L; and severe anemia, as less than 70 g/L.

**Main Outcomes and Measures:**

The main outcomes included 6 maternal outcomes (placental abruption, preterm birth, severe postpartum hemorrhage, shock, admission to the intensive care unit [ICU], and maternal mortality) and 3 neonatal outcomes (fetal growth restriction, malformation, and stillbirth). Multivariable logistic regression models were used to estimate the odds ratios (ORs) and 95% CIs of these outcomes among pregnant females with varying severity of anemia.

**Results:**

Among 18 948 443 pregnant females aged 15 to 49 years (mean [SD] age, 29.42 [4.87] years), 17.78% were diagnosed with anemia during pregnancy, including 9.04% with mild anemia, 2.62% with moderate anemia, 0.21% with severe anemia, and 5.90% with anemia of unknown severity. Compared with no anemia, anemia severity during pregnancy was associated with increased risks of placental abruption (mild: adjusted OR [aOR], 1.36 [95% CI, 1.34-1.38]; moderate: aOR, 1.98 [95% CI, 1.93-2.02]; severe: aOR, 3.35 [95% CI, 3.17-3.54]), preterm birth (mild: aOR, 1.08 [95% CI, 1.07-1.08]; moderate: aOR, 1.18 [95% CI, 1.17-1.19]; severe: aOR, 1.36 [95% CI, 1.32-1.41]), severe postpartum hemorrhage (mild: aOR, 1.45 [95% CI, 1.43-1.47]; moderate: aOR, 3.53 [95% CI, 3.47-3.60]; severe: 15.65 [95% CI, 15.10-16.22]), and fetal malformation (mild: aOR, 1.15 [95% CI, 1.14-1.17]; moderate: aOR, 1.19 [95% CI, 1.16-1.21]; severe: aOR, 1.62 [95% CI, 1.52-1.73]). Compared with no anemia, moderate or severe anemia were associated with increased risks of maternal shock (moderate: aOR, 1.50 [95% CI, 1.41-1.60]; severe: aOR, 14.98 [95% CI, 13.91-16.13]), ICU admission (moderate: aOR, 1.08 [95% CI, 1.01-1.16]; severe: aOR, 2.88 [95% CI, 2.55-3.25]), maternal death (moderate: aOR, 0.45 [95% CI, 0.30-0.65]; severe: aOR, 1.56 [95% CI, 0.97-2.48], fetal growth restriction (moderate: aOR, 0.80 [95% CI, 0.78-0.82]; severe: aOR, 1.08 [95% CI, 1.00-1.17]), and stillbirth (moderate: aOR,0.79 [95% CI, 0.76-0.81]; severe: aOR, 1.86 [95% CI, 1.75-1.98]), and mild anemia was associated with decreased risks (maternal shock: aOR, 0.67 [95% CI, 0.63-0.71]; ICU admission: aOR, 0.80 [95% CI, 0.76-0.84]; maternal death: aOR, 0.37 [95% CI, 0.29-0.49]; fetal growth restriction: aOR, 0.79 [95% CI, 0.77-0.80]; stillbirth: aOR, 0.59 [95% CI, 0.58-0.61]) after adjusting for sociodemographic characteristics and other complications during pregnancy.

**Conclusions and Relevance:**

The findings suggest that anemia during pregnancy is associated with maternal and fetal health outcomes and that mild anemia is associated with improved maternal and fetal survival and fetal growth. Further work is needed to validate the concentration of hemoglobin at which optimal maternal and fetal health are achieved.

## Key Points

**Question:**

Is severity of anemia during pregnancy associated with adverse obstetric outcomes?

**Findings:**

In this cohort study of 18 948 443 pregnant females, severity of anemia during pregnancy was associated with increased risk of placental abruption, preterm birth, severe postpartum hemorrhage, and fetal malformation. For maternal mortality, shock, and admission to the intensive care unit and fetal growth restriction and stillbirth, increased risks were observed among pregnant females with moderate or severe anemia and decreased risks among those with mild anemia.

**Meaning:**

In this cohort study, although severe anemia during pregnancy was associated with placenta-related morbidity, mild anemia was associated with decreased maternal and fetal mortality.

## Teaser

This cohort study used data from China’s Hospital Quality Monitoring System to investigate the association between severity of anemia during pregnancy and risks of maternal and fetal adverse outcomes.

## Introduction

Anemia is the most widespread nutritional deficiency among pregnant females in the world.^1^ According to the latest report of the World Health Organization, in most countries, the prevalence of anemia among pregnant and nonpregnant females aged 15 to 49 years increased from 2012 to 2016.^2^ The current worldwide progress is not on track in terms of achieving the nutrition target set by the 65th World Health Assembly, which is aiming for a 50% reduction in anemia prevalence among females of reproductive age by 2025.^3^ A total of 40.05% of pregnant females worldwide had anemia during pregnancy in 2016, with the highest prevalence (48.15%) in Southeast Asia.^4^ Because of the high prevalence of anemia, any adverse maternal and fetal outcomes associated with anemia during pregnancy would have a great public health impact.

The World Health Organization^5^ recommends the definition of severe, moderate, and mild anemia for pregnant women as hemoglobin concentrations of less than 70 g/L, 70 to 99, and 100 to 109 g/L (to convert g/L to g/dL, divide by 10.0), respectively. Although studies^6^ have reported adverse maternal and perinatal outcomes associated with anemia during pregnancy, the associations may vary when the severity of anemia is considered. Several studies^7,8^ reported similar or even lower risks of low birthweight and stillbirth in pregnant females with mild anemia compared with those who had normal hemoglobin concentrations. Some guidelines^9^ have recommended a hemoglobin cutoff lower than 110 g/L to define anemia during pregnancy; however, these recommendations still lack evidence and further work is needed to validate them. Furthermore, because of the large sample size requirements, little is known regarding the association between maternal anemia and rare adverse outcomes such as maternal death. Therefore, this study aimed to examine the association between the severity of anemia during pregnancy and the risk of adverse maternal and fetal outcomes through a retrospective cohort analysis of 18 948 443 pregnant females in China.

## Methods

All processes in this cohort study were reviewed and approved by the Peking University Third Hospital Medical Science Research Ethics Committee. The requirement for informed consent was waived because previously collected data containing no personally identifiable information were used. This study followed the Strengthening the Reporting of Observational Studies in Epidemiology (STROBE) reporting guideline.

### Data Source and Study Participants

The data were obtained from the Hospital Quality Monitoring System (HQMS). Established by the National Health Commission of China in 2012, the HQMS builds a patient-level national surveillance database by consistently collecting a data set using a standard protocol from the nationally standardized front page of all inpatient medical records across hospitals in 31 provinces of mainland China. During 2013 and 2019, the number of tertiary hospitals included in the HQMS increased from 823 to 1865, accounting for 46.1% and 67.8%, respectively, of all class 3 hospitals (hospitals with a capacity of ≥500 beds in China’s hierarchical health system, which classifies all hospitals as class 1, 2, or 3^10^) in the same year (eTables 1-3 in the Supplement). More than 600 variables were recorded in the HQMS database, including sociodemographic characteristics, admission and discharge diagnoses, procedures and operations, hospitalization expenses, and information about hospitals or divisions. The diagnoses were coded using the *International Statistical Classification of Diseases and Related Health Problems, Tenth Revision (ICD-10)* and the operations and procedures coded using the *International Classification of Diseases, Ninth Revision, Clinical Modification* (*ICD-9-CM*) by certified professional medical coders at each participating hospital.^11^ The HQMS performs automatic data quality control at the time of data submission to ensure data integrity, consistency, and accuracy. If inconsistencies are detected, the entire daily data package of the hospital is rejected and review and resubmission is requested.^12^

**Figure 1.**
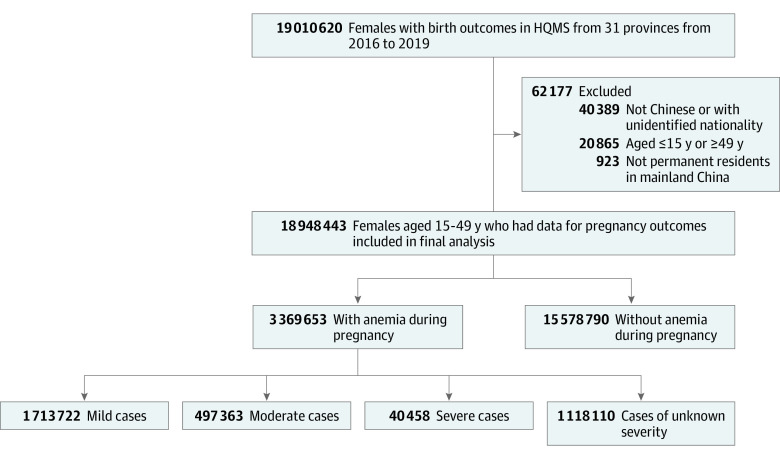
Flowchart of Study Participants HQMS indicates Hospital Quality Monitoring System.

In this study, we extracted data from all tertiary hospitals providing maternity services that were included in the HQMS from January 1, 2016, to December 31, 2019. The number of tertiary hospitals with maternity services in the HQMS increased from 1460 in 2016 to 1508 in 2019, accounting for approximately 60% of all tertiary hospitals in China. A total of 19 010 620 pregnant females seen at these hospitals had live births or stillbirths reported in the HQMS during the study period.^13^ Among these pregnant females, we excluded those who were not permanent residents of mainland China and were younger than 15 years or 50 years or older. The final cohort included 18 948 443 pregnant females, accounting for approximately one-third of all pregnancies in China over 4 years (Figure 1).^13^

### Definition of Variables

#### Exposure

Anemia during pregnancy (not puerperium), including mild anemia (hemoglobin concentration, 100-109 g/L), moderate anemia (hemoglobin concentration, 70-99 g/L), and severe anemia (hemoglobin concentration, <70 g/L), was identified using the relevant *ICD-10* codes (eTable 4 in the Supplement).^5^ Pregnant females with a diagnosis of anemia that could not be differentiated by severity with *ICD-10* codes were classified separately as having anemia of unknown severity.

#### Maternal and Fetal Outcomes

The maternal outcomes identified using *ICD-9-CM* or *ICD-10* codes included placental abruption, preterm birth (delivery before 37 weeks’ gestation), cesarean delivery, severe postpartum hemorrhage (females with *ICD-9-CM* or *ICD-10* codes of postpartum hemorrhage who were treated with blood transfusion or hysterectomy), shock, admission to the intensive care unit (ICU), and maternal death during hospital delivery. Fetal outcomes included growth restriction, malformation (congenital malformations, deformations, and chromosomal abnormalities diagnosed during hospitalization), and stillbirth (death before or during delivery after 20 weeks of pregnancy) (eTable 4 in the Supplement).

#### Complications During Pregnancy

Complications during pregnancy for which the time sequence relative to onset of anemia was unknown but that might have been associated with outcomes were identified as confounding variables using *ICD-9-CM* or *ICD-10* codes. These included in vitro fertilization, multiple pregnancies, hypertension disorders, diabetes, thyroid diseases, circulatory diseases, urinary diseases, respiratory diseases, digestive diseases, coagulation disorders, scarred uterus, placenta accreta spectrum, placenta previa, antepartum hemorrhage, intrauterine infection, abnormal amniotic fluid, cervical incompetence, and abnormal placenta.

### Statistical Analysis

We calculated the means and SDs for maternal age and used proportions to describe the distributions of geographic regions, year, marital status, medical insurance, maternal ethnicity, and complications during pregnancy by status and severity of anemia. The annual overall prevalence of anemia during pregnancy was calculated. We examined the association between maternal age and risk of anemia during pregnancy by performing a logistic regression adjusted for province, year, ethnicity, marital status, medical insurance information, in vitro fertilization status, and multiple pregnancy status. Maternal age was modeled using restricted cubic splines to allow for more flexibility of the association; knots were used according to the principle of minimized Akaike information criterion.^14^ Furthermore, we computed the adjusted odds of anemia during pregnancy at each maternal age and transformed the odds ratios (ORs) to estimate the absolute risks (probabilities) with 95% CIs.

We calculated the rate of each maternal and fetal outcome in pregnant females without anemia and in those with mild, moderate, and severe anemia. Multivariable logistic regression models were used to estimate the ORs and 95% CIs of these outcomes among pregnant females with varying severity of anemia during pregnancy. Sensitivity analyses were performed by adjusting for different covariates in multivariable models. In model A, we adjusted for maternal age, geographic region, year, marital status, medical insurance, maternal ethnicity, and complications during pregnancy. In model B, we additionally adjusted for complications during pregnancy for which the time sequence relative to onset of anemia was unknown but that might have been associated with the outcomes.

Taking into account the physiologic differences between females with singleton and multiple pregnancies, we additionally performed stratified analyses for all of the analyses and accounted for multiple pregnancies. All analyses were performed using SAS software, version 9.0 (SAS Institute Inc) and R, version 3.6.2 (R Project for Statistical Computing). Two-sided *P* < .05 was considered statistically significant.

## Results

A total of 18 948 443 pregnant females were included in the study (mean age [SD], 29.42 [4.87] years). Of these individuals, 91.76% were of Han ethnicity, 7.26% were members of other ethnic groups (includes >50 ethnic groups), and 0.98% were of unknown ethnicity; 1.97% had conceived through in vitro fertilization, and 3.46% had multiple pregnancies. Compared with pregnant females without anemia, those with anemia during pregnancy had a higher proportion of pregnancy complications except for cervical insufficiency (Table 1). The baseline maternal characteristics of those who had singleton and multiple pregnancies are detailed in eTable 5 in the Supplement.

**Table 1.** Maternal Baseline Characteristics by Anemia Status During Pregnancy^a^

Characteristic	Patients, No. (%)						
	No anemia (n = 15 578 790)	Anemia					Total (N = 18 948 443)
		Mild (n = 1 713 722)	Moderate (n = 497 363)	Severe (n = 40 458)	Unknown severity (n = 1 118 110)	All (n = 3 369 653)	
Region							
Eastern China	7 810 807 (50.14)	1 084 724 (63.30)	256 672 (51.61)	16 717 (41.32)	500 471 (44.76)	1 858 584 (55.16)	9 669 391 (51.03)
Central China	3 786 454 (24.31)	242 273 (14.14)	104 682 (21.05)	4927 (12.18)	221 113 (19.78)	572 995 (17.00)	4 359 449 (23.01)
Western China	3 981 529 (25.56)	386 725 (22.57)	136 009 (27.35)	18 814 (46.50)	396 526 (35.46)	938 074 (27.84)	4 919 603 (25.96)
Year							
2016	4 132 573 (26.53)	400 214 (23.35)	56 021 (11.26)	13 594 (33.60)	249 316 (22.30)	719 145 (21.34)	4 851 718 (25.60)
2017	4 098 427 (26.31)	452 813 (26.42)	77 360 (15.55)	9488 (23.45)	280 092 (25.05)	819 753 (24.33)	4 918 180 (25.96)
2018	3 775 312 (24.23)	452 237 (26.39)	100 164 (20.14)	6707 (16.58)	283 773 (25.38)	842 881 (25.01)	4 618 193 (24.37)
2019	3 572 478 (22.93)	408 458 (23.83)	263 818 (53.04)	10 669 (26.37)	304 929 (27.27)	987 874 (29.32)	4 560 352 (24.07)
Age, mean (SD), y	29.42 (4.89)	29.46 (5.13)	29.51 (5.35)	29.46 (6.11)	29.43 (5.19)	29.46 (5.19)	29.42 (4.87)
Ethnicity							
Han	14 340 366 (92.05)	1 566 686 (91.42)	449 605 (90.40)	27 477 (67.91)	1 002 603 (89.67)	3 046 371 (90.41)	17 386 737 (91.76)
Other^b^	1 083 293 (6.95)	136 694 (7.98)	43 485 (8.74)	12 848 (31.76)	99 462 (8.90)	292 489 (8.68)	1 375 782 (7.26)
Unknown	155 131 (1.00)	10 342 (0.60)	4273 (0.86)	133 (0.33)	16 045 (1.44)	30 793 (0.91)	185 924 (0.98)
Marital status							
Married	14 586 004 (93.63)	1 621 127 (94.60)	464 826 (93.46)	30 469 (75.31)	1 061 613 (94.95)	3 178 035 (94.31)	17 764 039 (93.75)
Unmarried	483 754 (3.11)	58 611 (3.42)	19 689 (3.96)	1652 (4.08)	31 931 (2.86)	111 883 (3.32)	595 637 (3.14)
Widowed or divorced	95 807 (0.61)	6802 (0.40)	1879 (0.38)	135 (0.33)	2782 (0.25)	11 598 (0.34)	107 405 (0.57)
Unknown	413 225 (2.65)	27 182 (1.59)	10 969 (2.21)	8202 (20.27)	21 784 (1.95)	68 137 (2.02)	481 362 (2.54)
Medical insurance							
Yes	10 829 503 (69.51)	1 116 359 (65.14)	311 216 (62.57)	19 235 (47.54)	778 018 (69.58)	2 224 828 (66.03)	13 054 331 (68.89)
No	4 743 215 (30.45)	597 115 (34.84)	186 128 (37.42)	21 221 (52.45)	339 960 (30.40)	1 144 424 (33.96)	5 887 639 (31.07)
Unknown	6072 (0.04)	248 (0.01)	19 (0.00)	2 (0.00)	132 (0.01)	401 (0.01)	6473 (0.03)
Maternal complications during pregnancy							
In vitro fertilization	295 701 (1.90)	36 613 (2.14)	16 825 (3.38)	993 (2.45)	22 525 (2.01)	76 956 (2.28)	372 657 (1.97)
Multiple pregnancies	486 602 (3.12)	82 407 (4.81)	34 762 (6.99)	2552 (6.31)	49 169 (4.40)	168 890 (5.01)	655 492 (3.46)
Hypertension disorders	889 798 (5.71)	94 068 (5.49)	34 648 (6.97)	5010 (12.38)	72 220 (6.46)	205 946 (6.11)	1 095 744 (5.78)
Diabetes	2 020 919 (12.97)	220 997 (12.90)	64 098 (12.89)	5372 (13.28)	148 103 (13.25)	438 570 (13.02)	2 459 489 (12.98)
Thyroid diseases	678 072 (4.35)	98 343 (5.74)	28 076 (5.64)	2004 (4.95)	68 203 (6.10)	196 626 (5.84)	874 698 (4.62)
Circulatory diseases	171 453 (1.10)	26 897 (1.57)	11 007 (2.21)	1172 (2.90)	17 160 (1.53)	56 236 (1.67)	227 689 (1.20)
Urinary diseases	79 868 (0.51)	18 734 (1.09)	6911 (1.39)	628 (1.55)	8136 (0.73)	34 409 (1.02)	114 277 (0.60)
Respiratory diseases	152 073 (0.98)	29 136 (1.70)	10 651 (2.14)	1381 (3.41)	17 463 (1.56)	58 631 (1.74)	210 704 (1.11)
Digestive diseases	340 055 (2.18)	56 105 (3.27)	21 528 (4.33)	1975 (4.88)	42 420 (3.79)	122 028 (3.62)	462 083 (2.44)
Coagulation disorders	30 547 (0.20)	26 225 (1.53)	19 118 (3.84)	1493 (3.69)	154 849 (13.85)	201 685 (5.99)	232 232 (1.23)
Scarred uterus	3 281 344 (21.06)	445 916 (26.02)	136 249 (27.39)	9524 (23.54)	280 909 (25.12)	872 598 (25.90)	4 153 942 (21.92)
Placenta previa	280 134 (1.80)	45 473 (2.65)	22 864 (4.60)	2370 (5.86)	32 902 (2.94)	103 609 (3.07)	383 743 (2.03)
Placenta accreta spectrum	326 700 (2.10)	43 897 (2.56)	22 257 (4.48)	2112 (5.22)	37 158 (3.32)	105 424 (3.13)	432 124 (2.28)
Antepartum hemorrhage	12 653 (0.08)	1769 (0.10)	752 (0.15)	186 (0.46)	1558 (0.14)	4265 (0.13)	16 918 (0.09)
Intrauterine infection	152 691 (0.98)	41 829 (2.44)	13 903 (2.80)	1722 (4.26)	11 878 (1.06)	69 332 (2.06)	222 023 (1.17)
Abnormal amniotic fluid	963 679 (6.19)	141 080 (8.23)	37 773 (7.59)	2156 (5.33)	57 201 (5.12)	238 210 (7.07)	1 201 889 (6.34)
Cervical incompetence	438 392 (2.81)	17 608 (1.03)	4609 (0.93)	292 (0.72)	18 195 (1.63)	40 704 (1.21)	479 096 (2.53)
Abnormal placenta	328 430 (2.11)	49 367 (2.88)	17 559 (3.53)	1124 (2.78)	27 091 (2.42)	95 141 (2.82)	423 571 (2.24)

SI conversion factor: To convert hemoglobin concentration to grams per deciliter, divide by 10.0.

^a^ No anemia was defined as a hemoglobin concentration of 110 g/L or greater; mild anemia, 100 to 109 g/L; moderate anemia, 70 to 99 g/L; severe anemia, less than 70 g/L.

^b^ Because there are more than 50 ethnic groups in China, other groups are not listed individually.

**Figure 2.**
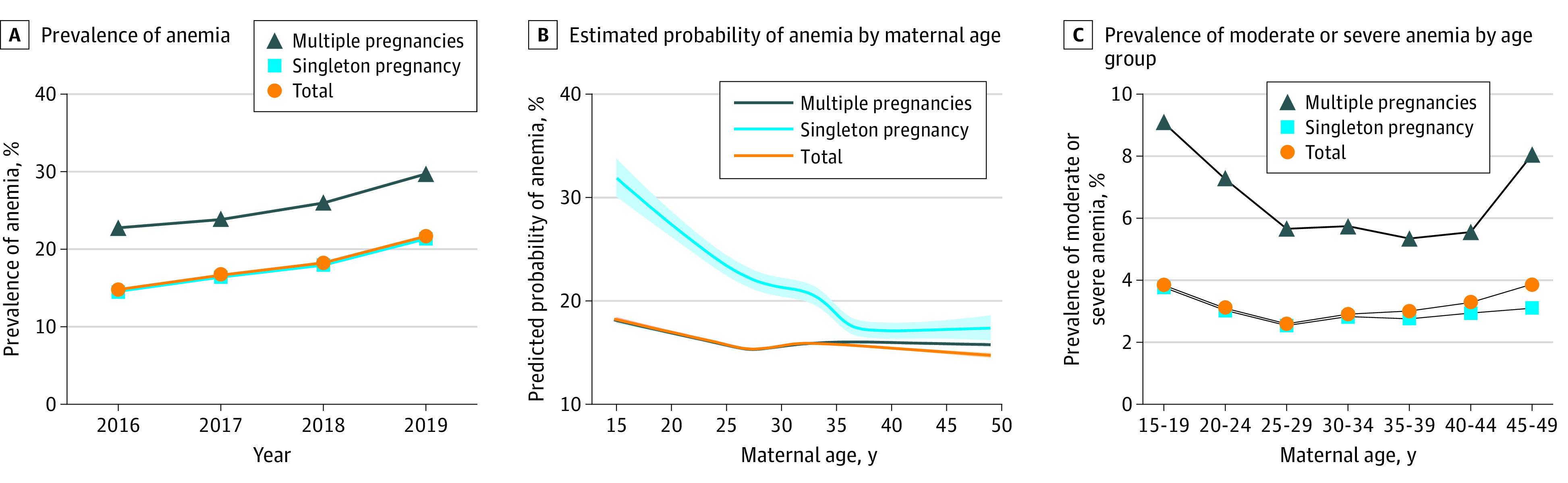
Rate of Anemia During Pregnancy by Year and Maternal Age

A total of 3 369 653 (17.78%) participants were diagnosed with anemia during pregnancy, including 1 713 722 (9.04%) with mild anemia, 497 363 (2.62%) with moderate anemia, 40 458 (0.21%) with severe anemia, and 1 118 110 (5.90%) with anemia of unknown severity (Figure 1). From 2016 to 2019, the prevalence of anemia during pregnancy increased from 14.82% to 21.66% (Figure 2A). The association between maternal age and the probability of anemia during pregnancy is presented as an L-shaped curve. The estimated probability of anemia during pregnancy decreased from 20.62% (95% CI, 20.38%-20.87%) at 15 years of age to 16.85% (95% CI, 16.85%-17.08%) at 28 years of age and then remained relatively stable. A similar trend was found for both singleton and multiple pregnancies (Figure 2B and C).

The prevalence of adverse maternal and fetal outcomes is shown in Table 2. Overall, compared with pregnant females without anemia, those with anemia during pregnancy had a higher risk of adverse outcomes except for admission to the ICU (adjusted OR [aOR], 0.89; 95% CI, 0.86-0.92), maternal death (aOR, 0.57; 95% CI, 0.49-0.67), fetal growth restriction (aOR, 0.85; 95% CI, 0.84-0.86), and stillbirth (aOR, 0.65; 95% CI, 0.64-0.66) after adjusting for sociodemographic characteristics and maternal complications during pregnancy (Table 3).

**Table 2.** Maternal and Fetal Adverse Outcomes by Severity of Anemia During Pregnancy^a^

Outcome	Patients, No. (%)						
	No anemia (n = 15 578 790)	Anemia					Total (N = 18 948 443)
		Mild (n = 1 713 722)	Moderate (n = 497 363)	Severe (n = 40 458)	Unknown severity (n = 1 118 110)	All (n = 3 369 653)	
**Maternal**							
Placental abruption	128 607 (0.83)	19 862 (1.16)	9275 (1.86)	1496 (3.70)	13 556 (1.21)	44 189 (1.31)	172 796 (0.91)
Preterm birth	864 698 (5.55)	118 470 (6.91)	42 725 (8.59)	4727 (11.68)	73 610 (6.58)	239 532 (7.11)	1 104 230 (5.83)
Severe postpartum hemorrhage	125 612 (0.81)	21 704 (1.27)	17 819 (3.58)	4822 (11.92)	25 392 (2.27)	69 737 (2.07)	195 349 (1.03)
Shock	12 610 (0.08)	1237 (0.07)	1180 (0.24)	1047 (2.59)	1980 (0.18)	5444 (0.16)	18 054 (0.10)
Admission to ICU	20 241 (0.13)	1690 (0.10)	948 (0.19)	333 (0.82)	2125 (0.19)	5096 (0.15)	25 337 (0.13)
Mortality, per 100 000 deliveries	1357 (8.71)	60 (3.50)	28 (5.63)	20 (49.43)	132 (11.81)	240 (7.12)	1597 (8.43)
Cesarean delivery	7 178 444 (46.08)	855 105 (49.90)	267 052 (53.69)	20 218 (49.97)	587 060 (52.50)	1 729 435 (51.32)	8 907 879 (47.01)
**Fetal**							
Fetal growth restriction	148 669 (0.95)	16 792 (0.98)	6052 (1.22)	776 (1.92)	11 890 (1.06)	35 510 (1.05)	184 179 (0.97)
Malformation	184 895 (1.19)	25 736 (1.50)	9010 (1.81)	954 (2.36)	16 471 (1.47)	52 171 (1.55)	237 066 (1.25)
Stillbirth	145 523 (0.93)	9799 (0.57)	4120 (0.83)	1097 (2.71)	7417 (0.66)	22 433 (0.67)	167 956 (0.89)

Abbreviation: ICU, intensive care unit.

SI conversion factor: To convert hemoglobin concentration to grams per deciliter, divide by 10.0.

^a^ No anemia was defined as a hemoglobin concentration of 110 g/L or greater; mild anemia, 100 to 109 g/L; moderate anemia, 70 to 99 g/L; severe anemia, less than 70 g/L.

**Table 3.** Adjusted Odds Ratios for Maternal and Fetal Adverse Outcomes Associated With Severity of Anemia During Pregnancy^a^

Outcome	Odds ratio (95% CI)									
	Model A^b^					Model B^c^				
	Mild anemia	Moderate anemia	Severe anemia	Anemia of unknown severity	All categories	Mild anemia	Moderate anemia	Severe anemia	Anemia of unknown severity	All categories
**Maternal**										
Placental abruption	1.39 (1.37-1.42)^d^	2.13 (2.09-2.18)^d^	4.29 (4.07-4.53)^d^	1.41 (1.39-1.44)^d^	1.54 (1.53-1.56)^d^	1.36 (1.34-1.38)^d^	1.98 (1.93-2.02)^d^	3.35 (3.17-3.54)^d^	1.37 (1.34-1.40)^d^	1.49 (1.47-1.50)^d^
Preterm birth	1.16 (1.15-1.17)^d^	1.39 (1.38-1.40)^d^	1.90 (1.84-1.96)^d^	1.15 (1.14-1.16)^d^	1.20 (1.20-1.21)^d^	1.08 (1.07-1.08)^d^	1.18 (1.17-1.19)^d^	1.36 (1.32-1.41)^d^	1.08 (1.07-1.09)^d^	1.10 (1.09-1.10)^d^
Severe postpartum hemorrhage	1.68 (1.66-1.71)^d^	4.68 (4.61-4.76)^d^	19.13 (18.52-19.76)^d^	2.73 (2.70-2.77)^d^	2.65 (2.62-2.67)^d^	1.45 (1.43-1.47)^d^	3.53 (3.47-3.60)^d^	15.65 (15.10-16.22)^d^	2.23 (2.20-2.27)^d^	2.17 (2.15-2.20)^d^
Shock	0.85 (0.80-0.90)^d^	2.65 (2.49-2.82)^d^	30.51 (28.51-32.65)^d^	2.09 (1.99-2.19)^d^	1.89 (1.83-1.96)^d^	0.67 (0.63-0.71)^d^	1.50 (1.41-1.60)^d^	14.98 (13.91-16.13)^d^	1.39 (1.32-1.47)^d^	1.29 (1.24-1.33)^d^
Admission to ICU	0.97 (0.92-1.02)	1.83 (1.71-1.96)^d^	7.65 (6.85-8.55)^d^	1.31 (1.25-1.37)^d^	1.29 (1.25-1.34)^d^	0.80 (0.76-0.84)^d^	1.08 (1.01-1.16)^e^	2.88 (2.55-3.25)^d^	0.82 (0.78-0.87)^d^	0.89 (0.86-0.92)^d^
Mortality	0.43 (0.33-0.56)^d^	0.73 (0.50-1.06)	4.44 (2.80-7.03)^d^	1.37 (1.14-1.64)^d^	0.87 (0.75-1.00)^e^	0.37 (0.29-0.49)^d^	0.45 (0.30-0.65)^d^	1.56 (0.97-2.48)	0.80 (0.65-0.98)^e^	0.57 (0.49-0.67)^d^
Cesarean delivery	1.33 (1.33-1.34)^d^	1.43 (1.42-1.44)^d^	1.22 (1.19-1.24)^d^	1.27 (1.26-1.27)^d^	1.32 (1.32-1.33)^d^	1.13 (1.13-1.14)^d^	1.16 (1.15-1.17)^d^	0.92 (0.90-0.95)^d^	1.16 (1.15-1.16)^d^	1.14 (1.14-1.15)^d^
**Fetal**										
Fetal growth restriction	0.85 (0.83-0.86)^d^	0.93 (0.91-0.95)^d^	1.53 (1.42-1.65)^d^	1.07 (1.05-1.09)^d^	0.94 (0.93-0.95)^d^	0.79 (0.77-0.80)^d^	0.80 (0.78-0.82)^d^	1.08 (1.00-1.17)	0.98 (0.96-1.00)^d^	0.85 (0.84-0.86)^d^
Malformation	1.18 (1.17-1.20)^d^	1.23 (1.21-1.26)^d^	1.72 (1.62-1.84)^d^	1.14 (1.12-1.16)^d^	1.18 (1.17-1.19)^d^	1.15 (1.14-1.17)^d^	1.19 (1.16-1.21)^d^	1.62 (1.52-1.73)^d^	1.12 (1.10-1.14)^d^	1.15 (1.14-1.17)^d^
Stillbirth	0.61 (0.60-0.62)^d^	0.87 (0.84-0.90)^d^	2.24 (2.10-2.38)^d^	0.67 (0.65-0.69)^d^	0.69 (0.68-0.70)^d^	0.59 (0.58-0.61)^d^	0.79 (0.76-0.81)^d^	1.86 (1.75-1.98)^d^	0.62 (0.61-0.64)^d^	0.65 (0.64-0.66)^d^

Abbreviations: ICU, intensive care unit; IVF, in vitro fertilization.

SI conversion factor: To convert hemoglobin concentration to grams per deciliter, divide by 10.0.

^a^ The group with no anemia was used as the reference. No anemia was defined as a hemoglobin concentration of 110 g/L or greater; mild anemia, 100 to 109 g/L; moderate anemia, 70 to 99 g/L; severe anemia, less than 70 g/L.

^b^ Model A adjusted for province, year, age, ethnicity, IVF, multiple pregnancy, medical insurance, and marital status.

^c^ Model B adjusted for all maternal complications during pregnancy in addition to covariates in model A.

^d^ *P* < .005.

^e^ *P* < .05.

According to the multivariable adjusted analyses, the associations between the severity of anemia during pregnancy and the risk of adverse maternal and fetal outcomes generally presented 2 patterns. The first was an upward curve with increased risks among pregnant females with more severe anemia compared with no anemia, including risk of placental abruption (mild: aOR, 1.36 [95% CI, 1.34-1.38]; moderate: aOR, 1.98 [95% CI, 1.93-2.02]; severe: aOR, 3.35 [95% CI, 3.17-3.54]), preterm birth (mild: aOR, 1.08 [95% CI, 1.07-1.08]; moderate: aOR, 1.18 [95% CI, 1.17-1.19]; severe: aOR, 1.36 [95% CI, 1.32-1.41]), severe postpartum hemorrhage (mild: aOR, 1.45 [95% CI, 1.43-1.47]; moderate: aOR, 3.53 [95% CI, 3.47-3.60]; severe: aOR, 15.65 [95% CI, 15.10-16.22]), and fetal malformation (mild: aOR, 1.15 [95% CI, 1.14-1.17]; moderate: aOR, 1.19 [95% CI, 1.16-1.21]; severe: aOR, 1.62 [95% CI, 1.52-1.73]). The second was a reverse J-shaped curve, with the lowest risk among pregnant females with mild anemia and higher risks among those with moderate or severe anemia compared with no anemia, including risk of shock (mild: aOR, 0.67 [95% CI, 0.63-0.71]; moderate: aOR, 1.50 [95% CI, 1.41-1.60]; severe: aOR, 14.98 [95% CI, 13.91-16.13]), ICU admission (mild: aOR, 0.80 [95% CI, 0.76-0.84]; moderate: aOR, 1.08 [95% CI, 1.01-1.16]; severe: aOR, 2.88 [95% CI, 2.55-3.25]), maternal death (mild: aOR, 0.37 [95% CI, 0.29-0.49]; moderate: aOR, 0.45 [95% CI, 0.30-0.65]; severe: aOR, 1.56 [95% CI, 0.97-2.48]), fetal growth restriction (mild: aOR, 0.79 [95% CI, 0.77-0.80]; moderate: aOR, 0.80 [95% CI, 0.78-0.82]; severe: aOR, 1.08 [95% CI, 1.00-1.17]), and stillbirth (mild: aOR, 0.59 [95% CI, 0.58-0.61]; moderate: aOR, 0.79 [95% CI, 0.76-0.81]; severe: aOR, 1.86 [95% CI, 1.75-1.98]). Adjusting for different covariates did not substantially affect the estimates, which were similar for models A and B (Table 3). Similar associations were found for both singleton and multiple pregnancies according to the results of the stratified analyses (eTables 6-8 in the Supplement).

## Discussion

### Principal Findings

Because the hospital delivery rate in China has reached 99.9%, our hospital-based sample was equivalent to a population-based sample, which accounted for approximately one-third of all pregnancies in China over 4 years.^13,15^ In this large cohort study, we revealed a high risk of anemia among pregnant females in China, especially among females aged 15 to 28 years. We also found an association between anemia during pregnancy and maternal and fetal outcomes, which differed among females with varying severity of anemia. Three of the adverse outcomes that we observed—placental abruption, preterm birth, and severe postpartum hemorrhage—were associated with anemia during pregnancy regardless of the severity. However, for some adverse outcomes, including maternal shock, admission to the ICU, and mortality and fetal growth restriction and stillbirth, increased risks were found among those with moderate or severe anemia, with a decreased risk associated with mild anemia compared with normal hemoglobin concentrations.

### Results in the Context of the Previous Literature

Epidemiologic characteristics of anemia during pregnancy highlight the need for a public health response in China. First, several data sources support the increasing trend of anemia in pregnant females in China.^16,17,18^ We estimated the overall rate of anemia to be 21.66% among pregnant females in 2019, which was higher than the prevalence (17.2%) estimated by the 2010-2012 Chinese Nutrition and Health Surveillance^16^ and the estimate of 19.84% for anemia prevalence among urban pregnant females reported in a cross-sectional survey in 2016.^17^ Because of the inclusion of pregnancies in tertiary hospitals, the sample in our study was skewed toward a more urban population with better nutrition and health characteristics. Based on these findings, anemia in Chinese pregnant females appears to have been of moderate public health significance in both rural and urban areas according to the World Health Organization criterion.^2^ Second, most studies from China and other resource-limited countries have reported an L-shaped or reverse J-shaped curve for the association between maternal age and risk of anemia during pregnancy, suggesting a greater risk among those younger than 25 years.^18,19,20^ An increase in prevalence of anemia was also found among adolescent girls in China from 2005 to 2014.^21,22,23^ Anemia among young women requires special attention, considering that they are the most likely to become pregnant in the population.^24,25,26^

Several factors may have contributed to the increase in prevalence of anemia over time in China. With the improvements in prenatal care and the increasing attention to maternal anemia, more pregnant females with anemia may be identified and given a clinical diagnosis. Second, dietary patterns in China have shifted substantially in the past few decades, characterized by a shifting nutritional environment that promotes greater consumption of high-calorie, nutrient-poor foods.^27^ Pregnant females in China are not routinely advised to take iron supplements. A study showed that approximately 70% of cases of anemia among pregnant females in China were associated with iron deficiency.^17^ In addition, pursuing a slender body type has become increasingly popular among women in recent years.^28^

Placental or delivery-related conditions, including placental abruption, have been well-documented factors associated with postpartum hemorrhage.^29^ Placental abruption is also associated with preterm birth.^30^ Consistent with previous findings,^6,31,32,33^ our study showed that pregnant females with anemia, regardless of severity level, were more likely to experience these adverse outcomes compared with pregnant females with normal hemoglobin levels. However, we found a reverse J-shaped association between the severity of anemia during pregnancy and maternal mortality and other adverse outcomes, such as shock and admission to the ICU, with the lowest risk among pregnant females with mild anemia. Similar findings were reported in a prospective cohort study in India and Pakistan,^34^ in which the lowest maternal mortality was found among pregnant females with hemoglobin concentrations of 100 to 109 g/L. For fetal outcomes, a higher risk for fetal malformation was found among pregnant females with anemia than among pregnant females without anemia in our study, whereas mild anemia during pregnancy was associated with decreased risks of fetal growth restriction and stillbirth. Although inconsistent reports exist,^7^ several previous studies^6,31,34,35,36,37,38^ support our findings on fetal outcomes.

The mechanisms underlying these associations are not yet clear. Anemia during pregnancy mainly results from the decrease in hemoglobin and erythrocyte synthesis owing to insufficient bioavailability of hematopoietic nutrients and a greater increase in plasma volume compared with red blood cell mass.^1,39,40^ There is increasing evidence that downregulation of hepcidin and upregulation of erythropoietin related to iron deficiency may have protective effects for the cardiovascular system and other organs.^41,42^ The decrease to a relatively low hemoglobin level during pregnancy likely reflects good plasma volume expansion, which reduces blood viscosity, increases uteroplacental blood flow and uteroplacental perfusion, and is often accompanied by cardiovascular changes, including increased cardiac output and decreased peripheral resistance.^39^ These adaptations may benefit maternal survival and facilitate fetal growth and development. In addition, compensatory responses by the placenta may also be beneficial for fetal growth and survival, occurring at the expense of optimum placental development and fetal health during postnatal life. Iron deficiency anemia was found to be associated with increased placental size and angiogenesis as well as upregulation of placental transfer systems,^40,43^ which may favor fetal oxygen and nutrient supplies. This would facilitate fetal growth and survival but increase the risk of placental abruption and postpartum hemorrhage. In rats, iron deficiency during pregnancy results in changes in lipid metabolism and obesity, high blood pressure, enlarged hearts, and compromised nephrogenesis in offspring,^40^ which might explain the increase in fetal malformations observed in pregnant females. Despite these adaptations, hemoglobin concentrations that are too low may exceed the capability of physiologic compensation and thus may be associated with adverse outcomes in pregnant females and fetuses. Previous studies suggest that early awareness of both high and low hemoglobin concentrations in the context of adverse pregnancy outcomes deserves increased attention.^6^

### Clinical and Research Implications

The Healthy China 2030 blueprint^44^ and the National Nutrition Plan 2017-2030^45^ aimed to reduce the prevalence of anemia among pregnant females to 10% by 2030 as a national nutrition target, with a series of plans involving continuous monitoring, evaluation, and quality assurance developed to achieve this. A study^46^ suggested that providing iron supplementation to pregnant females with hemoglobin levels of 100 g/L of greater who have good iron stores may be associated with an increase in preterm delivery and neonatal asphyxia. Based on our findings of the association of mild anemia with improved maternal and fetal survival, the use of a hemoglobin cutoff lower than 110 g/L to define anemia during pregnancy is suggested. Efforts to reduce the prevalence of anemia should identify the major determinants and then develop and implement an evidence-based intervention package, which usually depends on a high priority in national health policies, strategies, and plans and a coordinated multisectoral and multilevel response.^2^ We believe that interventions for moderate to severe anemia should be recommended and that monitoring and prevention of potential adverse outcomes are needed for pregnant females with anemia.

### Limitations

This study has several limitations. First, we identified anemia by severity using *ICD-10* codes and could not specify the type of anemia and the time when the hemoglobin concentration was measured during pregnancy. Second, despite controlling for many covariates in the multivariable adjusted analyses, we did not measure several important factors, especially iron supplementation and other nutritional conditions during pregnancy, which may have confounded the observed associations because of the retrospective nature of the HQMS data. Third, our study only included pregnant females seen in tertiary hospitals. To improve the representativeness of the sample and the generalization of the results, the HQMS should extend the sampling scope to hospitals across all levels.

## Conclusions

In this retrospective cohort study using HQMS data for 18 948 443 pregnant females in China from January 1, 2016, to December 31, 2019, we found that approximately 17.78% of pregnant females had anemia. The risk of anemia was higher among females aged 15 to 28 years. Although severe anemia during pregnancy was associated with placenta-related morbidity, mild anemia was associated with decreased maternal and fetal mortality. Further work is needed to validate the concentration of hemoglobin at which optimal maternal and fetal health are achieved. The findings suggest that interventions for moderate to severe anemia should be recommended but that low hemoglobin levels during pregnancy should be treated with caution until their effects on mothers and fetuses are understood.

## Notes

Supplement. **eTable 1.** Number of Class 3 Hospitals, Pregnancies and Livebirths in HQMS, 2013-2019 **eTable 2.** Number of Class 3 Hospitals With Obstetric Departments in HQMS, 2016-2019 **eTable 3.** Number of Pregnancies in HQMS in Each Province (According to the Delivery Hospital Location), 2016-2019 **eTable 4.** Variables and the ICD Codes **eTable 5.** Maternal Baseline Characteristics With Respect to Anemic Status During Pregnancy **eTable 6.** Maternal and Fetal Adverse Outcomes With Respect to Severity of Anemia During Pregnancy **eTable 7.** Adjusted ORs for Maternal and Fetal Adverse Outcomes According to Severity of Anemia During Pregnancy in Singleton Pregnancies **eTable 8.** Adjusted ORs for Maternal and Fetal Adverse Outcomes According to Severity of Anemia During Pregnancy in Multiple Pregnancies
