# Supplementary material for: Severity of Anemia During Pregnancy and Adverse Maternal and Fetal Outcomes
Source: JAMA Netw Open. 2022 Feb 3;5(2):e2147046. doi: 10.1001/jamanetworkopen.2021.47046 (PMC8814908; doi:10.1001/jamanetworkopen.2021.47046)
Supplement: Supplement. — eTable 1. Number of Class 3 Hospitals, Pregnancies and Livebirths in HQMS, 2013-2019 eTable 2. Number of Class 3 Hospitals With Obstetric Departments in HQMS, 2016-2019 eTable 3. Number of Pregnancies in HQMS in Each Province (According to the Delivery Hospital Location), 2016-2019 eTable 4. Variables and the ICD Codes eTable 5. Maternal Baseline Characteristics With Respect to Anemic Status During Pregnancy eTable 6. Maternal and Fetal Adverse Outcomes With Respect to Severity of Anemia During Pregnancy eTable 7. Adjusted ORs for Maternal and Fetal Adverse Outcomes According to Severity of Anemia During Pregnancy in Singleton Pregnancies eTable 8. Adjusted ORs for Maternal and Fetal Adverse Outcomes According to Severity of Anemia During Pregnancy in Multiple Pregnancies [file jamanetwopen-e2147046-s001.pdf]

Supplementary Online Content

Shi H, Chen L, Wang Y, et al. Severity of anemia during pregnancy and adverse maternal and fetal outcomes. *JAMA Netw Open*. 2022;5(2):e2147046. doi:10.1001/jamanetworkopen.2021.47046

eTable 1. Number of Class 3 Hospitals, Pregnancies and Livebirths in HQMS, 2013-2019
eTable 2. Number of Class 3 Hospitals With Obstetric Departments in HQMS, 2016-2019
eTable 3. Number of Pregnancies in HQMS in Each Province (According to the Delivery Hospital Location), 2016-2019
eTable 4. Variables and the ICD Codes
eTable 5. Maternal Baseline Characteristics With Respect to Anemic Status During Pregnancy
eTable 6. Maternal and Fetal Adverse Outcomes With Respect to Severity of Anemia During Pregnancy
eTable 7. Adjusted ORs for Maternal and Fetal Adverse Outcomes According to Severity of Anemia During Pregnancy in Singleton Pregnancies
eTable 8. Adjusted ORs for Maternal and Fetal Adverse Outcomes According to Severity of Anemia During Pregnancy in Multiple Pregnancies

This supplementary material has been provided by the authors to give readers additional information about their work.

eTable 1. Number of Class 3 hospitals, pregnancies and livebirths in HQMS, 2013-2019

	2013	2014	2015	2016	2017	2018	2019
No. of Class 3 hospitals all over the country (a)*	1 787	1 954	2 123	2 232	2 340	2 548	2 749
No. of livebirths all over the country (b)*	15 108 153	15 178 881	14 544 524	18 466 561	17 578 815	15 207 729	14 551 298
No. of Class 3 hospitals in HQMS (c)	823	907	920	1 810	1 835	1 853	1 865
Percentage (%) (d) (d=c/a*100%)	46.1	46.4	43.3	81.1	78.4	72.7	67.8
No. of Class 3 hospitals with obstetric departments in HQMS (e)	572	674	704	1 460	1 471	1 487	1 508
Percentage (%) (f) (f=e/c*100%)	69.5	74.3	76.5	80.7	80.2	80.2	80.9
No. of Pregnancies in HQMS (g)	895 711	1 641 632	1 554 459	4 851 718	4 918 180	4 618 193	4 560 352
Ratio (%) (h) (h=g/b*100%)	5.9	10.8	10.7	26.3	28.0	30.4	31.3

*Data were from National Health Commission of the People’s Republic of China. China Health Statistics Yearbook 2019. Beijing: Union Medical University Press; 2020.

© 2022 Shin H et al. *JAMA Network Open*.

eTable 2. Number of Class 3 hospitals with obstetric departments in HQMS, 2016-2019

**Province**	**2016**	**2017**	**2018**	**2019**
Anhui	40	41	40	44
Beijing	36	35	35	35
Chongqing	22	22	21	22
Fujian	37	38	39	37
Gansu	26	25	26	26
Guangdong	120	121	120	120
Guangxi	55	56	56	58
Guizhou	40	40	41	42
Hainan	15	15	15	16
Hebei	46	45	45	54
Henan	63	62	62	64
Heilongjiang	52	50	53	56
Hubei	70	72	70	71
Hunan	56	58	61	66
Jilin	30	30	30	30
Jiangsu	86	86	86	89
Jiangxi	46	46	46	47
Liaoning	70	71	73	72
Inner Mongolia	38	40	40	40
Ningxia	10	10	10	10
Qinghai	15	14	14	15
Shandong	77	77	81	81
Shanxi	40	40	40	40
Shaanxi	40	42	43	43
Shanghai	23	23	25	24
Sichuan	137	138	140	142
Tianjin	22	21	21	22
Xizang	5	7	7	9
Xinjiang	38	40	42	29
Yunnan	29	28	28	28
Zhejiang	76	78	77	76
**Total**	**1460**	**1471**	**1487**	**1508**

© 2022 Shin H et al. *JAMA Network Open*.

eTable 3. Number of pregnancies in HQMS in each province (according to the delivery hospital location), 2016-2019

**Province**	**2016**	**2017**	**2018**	**2019**
Anhui	146824	163298	155768	148230
Beijing	111740	102127	96881	108216
Chongqing	67329	69057	65718	70136
Fujian	186725	197964	171918	162987
Gansu	57001	57943	71575	47473
Guangdong	546798	599540	550467	552799
Guangxi	182109	199916	182356	172028
Guizhou	122673	150317	152740	141753
Hainan	49357	54402	51799	51948
Hebei	149161	139294	121927	149127
Henan	235883	222866	210042	198494
Heilongjiang	65070	58964	60190	57949
Hubei	179616	181579	173676	168855
Hunan	182876	191232	176519	175283
Jilin	70414	62588	57138	57579
Jiangsu	376488	358534	333831	317711
Jiangxi	134754	142585	129526	118670
Liaoning	140361	128396	123049	125349
Inner Mongolia	79028	71382	72816	82730
Ningxia	26688	24618	28427	33486
Qinghai	21979	22021	23571	27221
Shandong	418876	428345	387964	371083
Shanxi	113635	104053	105358	109935
Shaanxi	137212	140937	146296	144662
Shanghai	113648	101052	92301	93024
Sichuan	310816	304702	286410	293232
Tianjin	61355	62423	55375	59543
Xizang	6144	6514	10167	10960
Xinjiang	90126	84229	80024	75858
Yunnan	110876	120579	118760	117008
Zhejiang	356156	366723	325604	317023
**Total**	**4851718**	**4918180**	**4618193**	**4560352**

© 2022 Shin H et al. *JAMA Network Open*.

eTable 4. Variables and the ICD codes

Variables	ICD codes
Exposures	
Anemia during pregnancy	O99.000, O99.003, O99.004, O99.005, O99.006, O99.007
Mild anemia	O99.005
Moderate anemia	O99.006
Severe anemia	O99.007
Maternal outcomes	
Placental abruption	O45.000, O45.001, O45.800, O45.801, O45.900
Preterm birth	O60.000, O60.001, O60.100, O60.300, P07.300, P59.000, P61.200
Severe postpartum hemorrhage	O72.001, O72.002, O72.201, O72.101, O72.202, O72.000, O72.100, O72.200
Shock	O75.100, O75.101, R57.100, R57.101
Cesarean delivery	O66.401, O82.000, O82.100, O82.200, O82.800, O82.900, O84.200, P03.401, O86.002, O90.000, P03.400, 74.0 001, 74.1 001, 74.1 002, 74.1 003, 74.2 002, 74.4 003
Fetal outcomes	
Fetal growth restriction (FGR)	O36.500, O36.504, O36.503, P05.000, P05.100, P05.101, P05.102, P05.200, P05.201, P05.900
Malformation	O35.000, O35.001, O35.002, O35.003, O35.004, O35.005, O35.006, O35.007, O35.008, O35.009, O35.100, O35.101, O35.102, O35.200, O35.201, O35.202, O35.203, O35.204, O35.205, O35.206, O28.001, O35.800, O35.801, O35.802, O35.803, O35.804, O35.805, O35.806, O35.807, O35.808, O35.809, O35.810, O35.811, O35.812, O35.813, O35.814, O35.815, O35.816, O35.817, O35.818, O35.819, O35.820, O35.821, O35.822, O35.823, O35.900, O36.200, O36.201
Stillbirth	O36.400, O36.401, O31.200, O31.201, P95.x00, Z37.100, Z37.300, Z37.302, Z37.303, Z37.601, Z37.400, Z37.700
Maternal complications during pregnancy	
In vitro fertilization (IVF)	Z31.200, Z31.201, Z37.002, Z37.204, Z37.303, Z37.502, 69.9200x004, 69.9200x006, 69.9202
Multiple pregnancies	O30.000, O30.100, O30.200, O30.800, O30.801, O30.900, O31.000, O31.100, O31.200, O31.201, O31.800, O32.500, O32.501, O35.811, O43.001, O63.200, O63.201, O66.100, O66.101, O69.209, O84.000, O84.100, O84.200, O84.800, O84.900, P01.500, P01.501, P01.502, P02.301, P50.300, P50.500, Z37.200, Z37.201, Z37.202, Z37.203, Z37.204, Z37.300, Z37.301, Z37.302, Z37.303, Z37.400, Z37.500, Z37.501, Z37.502, Z37.600, Z37.601, Z37.700, Z38.300, Z38.400, Z38.500, Z38.600, Z38.700, Z38.800

© 2022 Shin H et al. *JAMA Network Open*.

Variables	ICD codes
Hypertension disorders	O10.000, O10.001, O10.400, O10.401, O10.900, O16.x00, O13.x00, O13.x01, O11.x00, O11.x01, O13.x02, O14.000, O14.100, O14.101, O14.102, O14.900, O15.000, O15.001, O15.900, O10.100, O10.101, O10.200, O10.201, O10.300, O10.301
Diabetes	O24.000, O24.100, O24.200, O24.300, O24.301, O24.400, O24.900
Thyroid diseases	O99.217, O99.216, O99.215, O99.218, O99.219
Circulatory diseases	O10.100, O10.101, O10.300, O10.301, O99.400, O99.419, O99.420, O99.428, O99.429, O99.432, O99.430, O99.431, O99.433, O99.409, O99.415, O99.408, O99.421, O99.413, O99.416, O99.403, O99.418, O99.422, O99.412, O99.410, O99.423, O99.424, O99.406, O99.407, O99.411, O99.404, O99.405
Urinary diseases	O26.801, O26.803, O26.804, O26.805, O23.000, O23.001, O23.100, O23.101, O23.200, O23.300, O23.400, O23.900, O23.901, O99.806
Respiratory diseases	O99.501, O99.502, O99.503, O99.504, O99.505, O99.506, O99.510, O99.511, O99.512, O99.508, O99.500
Digestive diseases	O99.600, O99.601, O99.602, O99.603, O99.604, O99.605, O99.606, O99.607, O99.608, O99.609, O99.610, O99.611, O99.612, O99.613, O99.614, O99.615, O99.616, O99.617, O99.618, O99.619, O99.620, O99.621, O99.622, O99.623, O99.624, O26.600, O26.601, O26.604, O26.605, O26.606, O26.607
Coagulation disorders	O99.002, O99.101, O99.102, O99.103, O99.104, O99.105, O99.106, O99.107, O99.108, O99.100
Scarred uterus	N85.801, O00.807, O34.200, O34.201, O75.700
Placenta previa	O44.000, O44.001, O44.002, O44.003, O44.100, O44.101, O44.102, O44.103
Placenta accreta spectrum	O72.001
Antepartum hemorrhage	O46.000, O46.001, O46.800, O46.801, O46.900
Intrauterine infection	O41.100, O41.101, O41.102, O41.103, O41.104
Abnormal amniotic fluid	O40.x00, O41.000, O41.800
Cervical incompetence	O34.301, O34.300, N85.801
Abnormal placenta	O43.100, O43.101, O43.102, O43.103, O43.104, O43.105, O43.106, O43.107, O43.108, O43.109, O43.110, O43.111, O43.112, O43.800, O43.801, O43.802, O43.803, O43.804, O43.805, O43.900

© 2022 Shin H et al. *JAMA Network Open*.

eTable 5. Maternal baseline characteristics with respect to anemic status during pregnancy

	Singleton pregnancies							Multiple pregnancies						
	Non-anemia	Anemia					Total	Non-anemia	Anemia					Total
		Mild	Moderate	Severe	unknown severity	Subtotal			Mild	Moderate	Severe	unknown severity	Subtotal	
**No. of participants**	15092188	1631315	462601	37906	1068941	3200763	18292951	486602	82407	34762	2552	49169	168890	655492
**Region, No. (%)**														
Eastern China	7521858 (49.84)	1028392 (63.04)	236054 (51.03)	15447 (40.75)	474743 (44.41)	1754636 (54.82)	9276494 (50.71)	288949 (59.38)	56332 (68.36)	20618 (59.31)	1270 (49.76)	25728 (52.33)	103948 (61.55)	392897 (59.94)
Central China	3695931 (24.49)	232545 (14.26)	99073 (21.42)	4626 (12.20)	212797 (19.91)	549041 (17.15)	4244972 (23.21)	90523 (18.60)	9728 (11.80)	5609 (16.14)	301 (11.79)	8316 (16.91)	23954 (14.18)	114477 (17.46)
Western China	3874399 (25.67)	370378 (22.70)	127474 (27.56)	17833 (47.05)	381401 (35.68)	897086 (28.03)	4771485 (26.08)	107130 (22.02)	16347 (19.84)	8535 (24.55)	981 (38.44)	15125 (30.76)	40988 (24.27)	148118 (22.60)
**Year, No. (%)**														
2016	4028454 (26.69)	383246 (23.49)	52372 (11.32)	13054 (34.44)	239840 (22.44)	688512 (21.51)	4716966 (25.79)	104119 (21.40)	16968 (20.59)	3649 (10.50)	540 (21.16)	9476 (19.27)	30633 (18.14)	134752 (20.56)
2017	3972087 (26.32)	431000 (26.42)	72036 (15.57)	9001 (23.75)	268268 (25.10)	780305 (24.38)	4752392 (25.98)	126340 (25.96)	21813 (26.47)	5324 (15.32)	487 (19.08)	11824 (24.05)	39448 (23.36)	165788 (25.29)
2018	3647837 (24.17)	428675 (26.28)	92632 (20.02)	6200 (16.36)	270779 (25.33)	798286 (24.94)	4446123 (24.31)	127475 (26.20)	23562 (28.59)	7532 (21.67)	507 (19.87)	12994 (26.43)	44595 (26.40)	172070 (26.25)
2019	3443810 (22.82)	388394 (23.81)	245561 (53.08)	9651 (25.46)	290054 (27.13)	933660 (29.17)	4377470 (23.93)	128668 (26.44)	20064 (24.35)	18257 (52.52)	1018 (39.89)	14875 (30.25)	54214 (32.10)	182882 (27.90)
**Age (years), mean (SD)**	29.30 (4.78)	29.27 (4.86)	29.26 (4.96)	29.29 (5.24)	29.31 (4.80)	29.28 (4.86)	29.30 (4.80)	33.10 (5.54)	33.21 (5.60)	32.80 (5.82)	31.91 (5.88)	32.09 (5.61)	32.78 (5.68)	33.02 (5.58)
**Ethnic origin, No. (%)**														
Han	13892789 (92.05)	1492649 (91.50)	418441 (90.45)	25521 (67.33)	958269 (89.65)	2894880 (90.44)	16787669 (91.77)	447577 (91.98)	74037 (89.84)	31164 (89.65)	1956 (76.65)	44334 (90.17)	151491 (89.70)	599068 (91.39)

© 2022 Shin H et al. *JAMA Network Open*.

Other	1047044 (6.94)	128683 (7.89)	40041 (8.66)	12261 (32.35)	95041 (8.89)	276026 (8.62)	1323070 (7.23)	36249 (7.45)	8011 (9.72)	3444 (9.91)	587 (23.00)	4421 (8.99)	16463 (9.75)	52712 (8.04)
Unknown	152355 (1.01)	9983 (0.61)	4119 (0.89)	124 (0.33)	15631 (1.46)	29857 (0.93)	182212 (1.00)	2776 (0.57)	359 (0.44)	154 (0.44)	9 (0.35)	414 (0.84)	936 (0.55)	3712 (0.57)
**Marital status, No. (%)**														
Married	14122860 (93.58)	1542020 (94.53)	432073 (93.40)	28256 (74.54)	1014530 (94.91)	3016879 (94.25)	17139739 (93.70)	463144 (95.18)	79107 (96.00)	32753 (94.22)	2213 (86.72)	47083 (95.76)	161156 (95.42)	624300 (95.24)
Unmarried	474327 (3.14)	57028 (3.50)	18723 (4.05)	1546 (4.08)	30897 (2.89)	108194 (3.38)	582521 (3.18)	9427 (1.94)	1583 (1.92)	966 (2.78)	106 (4.15)	1034 (2.10)	3689 (2.18)	13116 (2.00)
Widowed/ divorced	94116 (0.62)	6523 (0.40)	1741 (0.38)	124 (0.33)	2675 (0.25)	11063 (0.35)	105179 (0.57)	1691 (0.35)	279 (0.34)	138 (0.40)	11 (0.43)	107 (0.22)	535 (0.32)	2226 (0.34)
Unknown	400885 (2.66)	25744 (1.58)	10064 (2.18)	7980 (21.05)	20839 (1.95)	64627 (2.02)	465512 (2.54)	12340 (2.54)	1438 (1.74)	905 (2.60)	222 (8.70)	945 (1.92)	3510 (2.08)	15850 (2.42)
**Medical insurance, No. (%)**														
Yes	10491587 (69.52)	1065392 (65.31)	289937 (62.68)	17864 (47.13)	744458 (69.64)	2117651 (66.16)	12609238 (68.93)	337916 (69.44)	50967 (61.85)	21279 (61.21)	1371 (53.72)	33560 (68.25)	107177 (63.46)	445093 (67.90)
No	4594717 (30.44)	565742 (34.68)	172652 (37.32)	20040 (52.87)	324361 (30.34)	1082795 (33.83)	5677512 (31.04)	148498 (30.52)	31373 (38.07)	13476 (38.77)	1181 (46.28)	15599 (31.73)	61629 (36.49)	210127 (32.06)
Unknown	5884 (0.04)	181 (0.01)	12 (0.00)	2 (0.01)	122 (0.01)	317 (0.01)	6201 (0.03)	188 (0.04)	67 (0.08)	7 (0.02)	0 (0.00)	10 (0.02)	84 (0.05)	272 (0.04)
**Maternal complications during pregnancy, No. (%)**														
In vitro fertilization	206776 (1.37)	22126 (1.36)	8995 (1.94)	469 (1.24)	13776 (1.29)	45366 (1.42)	252142 (1.38)	88925 (18.27)	14487 (17.58)	7830 (22.52)	524 (20.53)	8749 (17.79)	31590 (18.70)	120515 (18.39)
Hypertension disorders	828114 (5.49)	83186 (5.10)	28962 (6.26)	4328 (11.42)	64109 (6.00)	180585 (5.64)	1008699 (5.51)	61684 (12.68)	10882 (13.21)	5686 (16.36)	682 (26.72)	8111 (16.50)	25361 (15.02)	87045 (13.28)
Diabetes	1917819	204826	57483	4960	138590	405859	2323678	103100	16171	6615	412	9513	32711	135811

© 2022 Shin H et al. *JAMA Network Open*.

	(12.71)	(12.56)	(12.43)	(13.08)	(12.97)	(12.68)	(12.70)	(21.19)	(19.62)	(19.03)	(16.14)	(19.35)	(19.37)	(20.72)
Thyroid diseases	649519 (4.30)	92612 (5.68)	25798 (5.58)	1855 (4.89)	64550 (6.04)	184815 (5.77)	834334 (4.56)	28553 (5.87)	5731 (6.95)	2278 (6.55)	149 (5.84)	3653 (7.43)	11811 (6.99)	40364 (6.16)
Circulatory diseases	162146 (1.07)	24696 (1.51)	9605 (2.08)	1020 (2.69)	15762 (1.47)	51083 (1.60)	213229 (1.17)	9307 (1.91)	2201 (2.67)	1402 (4.03)	152 (5.96)	1398 (2.84)	5153 (3.05)	14460 (2.21)
Urinary diseases	74598 (0.49)	17157 (1.05)	6089 (1.32)	547 (1.44)	7413 (0.69)	31206 (0.97)	105804 (0.58)	5270 (1.08)	1577 (1.91)	822 (2.36)	81 (3.17)	723 (1.47)	3203 (1.90)	8473 (1.29)
Respiratory diseases	144742 (0.96)	27195 (1.67)	9475 (2.05)	1226 (3.23)	16288 (1.52)	54184 (1.69)	198926 (1.09)	7331 (1.51)	1941 (2.36)	1176 (3.38)	155 (6.07)	1175 (2.39)	4447 (2.63)	11778 (1.80)
Digestive diseases	316350 (2.10)	51553 (3.16)	18936 (4.09)	1747 (4.61)	38605 (3.61)	110841 (3.46)	427191 (2.34)	23705 (4.87)	4552 (5.52)	2592 (7.46)	228 (8.93)	3815 (7.76)	11187 (6.62)	34892 (5.32)
Coagulation disorders	28579 (0.19)	24719 (1.52)	18082 (3.91)	1383 (3.65)	147821 (13.83)	192005 (6.00)	220584 (1.21)	1968 (0.40)	1506 (1.83)	1036 (2.98)	110 (4.31)	7028 (14.29)	9680 (5.73)	11648 (1.78)
Scarred uterus	3156957 (20.92)	421835 (25.86)	126714 (27.39)	8971 (23.67)	269034 (25.17)	826554 (25.82)	3983511 (21.78)	124387 (25.56)	24081 (29.22)	9535 (27.43)	553 (21.67)	11875 (24.15)	46044 (27.26)	170431 (26.00)
Placenta previa	266622 (1.77)	42346 (2.60)	20865 (4.51)	2171 (5.73)	31070 (2.91)	96452 (3.01)	363074 (1.98)	13512 (2.78)	3127 (3.79)	1999 (5.75)	199 (7.80)	1832 (3.73)	7157 (4.24)	20669 (3.15)
Placenta accreta spectrum	310026 (2.05)	40913 (2.51)	20203 (4.37)	1911 (5.04)	34753 (3.25)	97780 (3.05)	407806 (2.23)	16674 (3.43)	2984 (3.62)	2054 (5.91)	201 (7.88)	2405 (4.89)	7644 (4.53)	24318 (3.71)
Antepartum hemorrhage	12026 (0.08)	1652 (0.10)	694 (0.15)	170 (0.45)	1477 (0.14)	3993 (0.12)	16019 (0.09)	627 (0.13)	117 (0.14)	58 (0.17)	16 (0.63)	81 (0.16)	272 (0.16)	899 (0.14)
Intrauterine infection	146592 (0.97)	39795 (2.44)	12897 (2.79)	1606 (4.24)	11153 (1.04)	65451 (2.04)	212043 (1.16)	6099 (1.25)	2034 (2.47)	1006 (2.89)	116 (4.55)	725 (1.47)	3881 (2.30)	9980 (1.52)
Abnormal amniotic fluid	938641 (6.22)	135959 (8.33)	35504 (7.67)	2008 (5.30)	55096 (5.15)	228567 (7.14)	1167208 (6.38)	25038 (5.15)	5121 (6.21)	2269 (6.53)	148 (5.80)	2105 (4.28)	9643 (5.71)	34681 (5.29)
Cervical incompetence	424988 (2.82)	16463 (1.01)	4060 (0.88)	269 (0.71)	17271 (1.62)	38063 (1.19)	463051 (2.53)	13404 (2.75)	1145 (1.39)	549 (1.58)	23 (0.90)	924 (1.88)	2641 (1.56)	16045 (2.45)

© 2022 Shin H et al. *JAMA Network Open*.

Abnormal placenta	310025<br>(2.05)	45494<br>(2.79)	15621<br>(3.38)	1005<br>(2.65)	25116<br>(2.35)	87236<br>(2.73)	397261<br>(2.17)	18405<br>(3.78)	3873<br>(4.70)	1938<br>(5.58)	119<br>(4.66)	1975<br>(4.02)	7905<br>(4.68)	26310<br>(4.01)

© 2022 Shin H et al. *JAMA Network Open*.

eTable 6. Maternal and fetal adverse outcomes with respect to severity of anemia during pregnancy

	Singleton pregnancies							Multiple pregnancies						
	Non-anemia	Anemia					Total	Non-anemia	Anemia					Total
		Mild	Moderate	Severe	Unknown severity	Subtotal			Mild	Moderate	Severe	Unknown severity	Subtotal	
**No. of participants**	15092188	1631315	462601	37906	1068941	3200763	18292951	486602	82407	34762	2552	49169	168890	655492
**Maternal adverse outcomes, No. (%)**														
Placental abruption	122819(0.81)	18607 (1.14)	8581 (1.85)	1407 (3.71)	12877 (1.20)	41472 (1.30)	164291(0.90)	5788 (1.19)	1255 (1.52)	694 (2.00)	89 (3.49)	679 (1.38)	2717 (1.61)	8505 (1.30)
Preterm birth	743137 (4.92)	96243 (5.90)	32225 (6.97)	3772 (9.95)	58430 (5.47)	190670 (5.96)	933807 (5.10)	121561 (24.98)	22227 (26.97)	10500 (30.21)	955 (37.42)	15180 (30.87)	48862 (28.93)	170423 (26.00)
Severe postpartum hemorrhage	114566 (0.76)	19219 (1.18)	15584 (3.37)	4297 (11.34)	22806 (2.13)	61906 (1.93)	176472 (0.96)	11046 (2.27)	2485 (3.02)	2235 (6.43)	525 (20.57)	2586 (5.26)	7831 (4.64)	18877 (2.88)
Shock	11845 (0.08)	1130 (0.07)	1076 (0.23)	938 (2.47)	1801 (0.17)	4945 (0.15)	16790 (0.09)	765 (0.16)	107 (0.13)	104 (0.30)	109 (4.27)	179 (0.36)	499 (0.30)	1264 (0.19)
Admission into ICU	19100 (0.13)	1558 (0.10)	844 (0.18)	300 (0.79)	1914 (0.18)	4616 (0.14)	23716 (0.13)	1141 (0.23)	132 (0.16)	104 (0.30)	33 (1.29)	211 (0.43)	480 (0.28)	1621 (0.25)
Maternal death (Per 100000 deliveries)	1312 (8.69)	59 (3.62)	28 (6.05)	20 (52.76)	127 (11.88)	234 (7.31)	1546 (8.45)	45 (9.25)	1 (1.21)	0 (0.00)	0 (0.00)	5 (10.17)	6 (3.55)	51 (7.78)
Cesarean delivery	6819014 (45.18)	791787 (48.54)	239154 (51.70)	18240 (48.12)	546952 (51.17)	1596133 (49.87)	8415147 (46.00)	359430 (73.87)	63318 (76.84)	27898 (80.25)	1978 (77.51)	40108 (81.57)	133302 (78.93)	492732 (75.17)
**Fetal adverse outcomes, No. (%)**														
Fetal growth restriction	129969 (0.86)	13339 (0.82)	4296 (0.93)	655 (1.73)	9619 (0.90)	27909 (0.87)	157878 (0.86)	18700 (3.84)	3453 (4.19)	1756 (5.05)	121 (4.74)	2271 (4.62)	7601 (4.50)	26301 (4.01)
Malformation	169829 (1.13)	23028 (1.41)	7617 (1.65)	869 (2.29)	14873 (1.39)	46387 (1.45)	216216 (1.18)	15066 (3.10)	2708 (3.29)	1393 (4.01)	85 (3.33)	1598 (3.25)	5784 (3.42)	20850 (3.18)
Stillbirth	131453 (0.87)	8101 (0.50)	3341 (0.72)	978 (2.58)	6362 (0.60)	18782 (0.59)	150235 (0.82)	14070 (2.89)	1698 (2.06)	779 (2.24)	119 (4.66)	1055 (2.15)	3651 (2.16)	17721 (2.70)

© 2022 Shin H et al. *JAMA Network Open*.

eTable 7. Adjusted ORs for maternal and fetal adverse outcomes according to severity of anemia during pregnancy in singleton pregnancies[a]

	Model A [OR (95% CI)][b]					Model B [OR (95% CI)][c]				
	Mild anemia [Hb: 100–109 g/L]	Moderate anemia [Hb: 70–99 g/L]	Severe anemia [Hb: <70 g/L]	Anemia with unknown severity	Overall anemia [Hb: <110 g/L]	Mild anemia [Hb: 100–109 g/L]	Moderate anemia [Hb: 70–99 g/L]	Severe anemia [Hb: <70 g/L]	Anemia with unknown severity	Overall anemia [Hb: <110 g/L]
**Maternal adverse outcomes**										
Placental abruption	1.40 (1.38, 1.42)[**]	2.18 (2.13, 2.23)[**]	4.44 (4.21, 4.69)[**]	1.43 (1.40, 1.45)[**]	1.56 (1.54, 1.58)[**]	1.37 (1.35, 1.39)[**]	2.03 (1.98, 2.07)[**]	3.47 (3.28, 3.67)[**]	1.39 (1.36, 1.41)[**]	1.50 (1.48, 1.52)[**]
Preterm birth	1.15 (1.15, 1.16)[**]	1.39 (1.38, 1.41)[**]	1.91 (1.84, 1.97)[**]	1.12 (1.12, 1.13)[**]	1.19 (1.18, 1.19)[**]	1.06 (1.05, 1.07)[**]	1.16 (1.15, 1.18)[**]	1.34 (1.29, 1.39)[**]	1.05 (1.04, 1.06)[**]	1.08 (1.07, 1.08)[**]
Severe postpartum hemorrhage	1.70 (1.67, 1.72)[**]	4.88 (4.80, 4.97)[**]	19.91 (19.25, 20.60)[**]	2.77 (2.73, 2.81)[**]	2.69 (2.66, 2.72)[**]	1.45 (1.43, 1.47)[**]	3.63 (3.56, 3.70)[**]	16.38 (15.77, 17.01)[**]	2.25 (2.21, 2.29)[**]	2.19 (2.17, 2.22)[**]
Shock	0.85 (0.80, 0.90)[**]	2.75 (2.58, 2.94)[**]	30.79 (28.66, 33.07)[**]	2.06 (1.95, 2.16)[**]	1.89 (1.83, 1.96)[**]	0.67 (0.63, 0.71)[**]	1.54 (1.44, 1.64)[**]	14.95 (13.82, 16.17)[**]	1.37 (1.30, 1.45)[**]	1.28 (1.23, 1.33)[**]
Admission into ICU	0.98 (0.93, 1.03)	1.88 (1.75, 2.01)[**]	7.75 (6.90, 8.72)[**]	1.29 (1.23, 1.35)[**]	1.29 (1.25, 1.34)[**]	0.80 (0.76, 0.85)[**]	1.11 (1.03, 1.20)[**]	2.89 (2.54, 3.28)[**]	0.81 (0.76, 0.85)[**]	0.89 (0.86, 0.92)[**]
Maternal death	0.45 (0.34, 0.58)[**]	0.78 (0.54, 1.14)	4.84 (3.06, 7.67)[**]	1.38 (1.14, 1.65)[**]	0.89 (0.77, 1.03)	0.39 (0.30, 0.51)[**]	0.48 (0.33, 0.70)[**]	1.67 (1.04, 2.67)[*]	0.80 (0.65, 0.99)[*]	0.59 (0.50, 0.69)[**]
Cesarean delivery	1.33 (1.32, 1.33)[**]	1.42 (1.41, 1.43)[**]	1.20 (1.18, 1.23)[**]	1.26 (1.26, 1.27)[**]	1.32 (1.31, 1.32)[**]	1.13 (1.12, 1.13)[**]	1.14 (1.13, 1.15)[**]	0.90 (0.88, 0.93)[**]	1.15 (1.14, 1.15)[**]	1.13 (1.13, 1.14)[**]
**Fetal adverse outcomes**										
Fetal growth restriction	0.80 (0.79, 0.82)[**]	0.86 (0.84, 0.89)[**]	1.66 (1.53, 1.79)[**]	1.05 (1.02, 1.07)[**]	0.90 (0.89, 0.91)[**]	0.74 (0.73, 0.76)[**]	0.74 (0.72, 0.77)[**]	1.16 (1.07, 1.26)[**]	0.96 (0.94, 0.98)[**]	0.81 (0.80, 0.82)[**]
Malformation	1.20 (1.19, 1.22)[**]	1.25 (1.22, 1.28)[**]	1.85 (1.73, 1.98)[**]	1.15 (1.13, 1.17)[**]	1.20 (1.19, 1.21)[**]	1.17 (1.16, 1.19)[**]	1.20 (1.18, 1.23)[**]	1.74 (1.62, 1.86)[**]	1.14 (1.12, 1.16)[**]	1.17 (1.16, 1.19)[**]
Stillbirth	0.59 (0.57, 0.60)[**]	0.87 (0.84, 0.90)[**]	2.32 (2.17, 2.48)[**]	0.66 (0.64, 0.68)[**]	0.68 (0.67, 0.69)[**]	0.57 (0.56, 0.59)[**]	0.79 (0.76, 0.82)[**]	1.93 (1.80, 2.06)[**]	0.61 (0.59, 0.62)[**]	0.64 (0.63, 0.65)[**]

[a] Non-anemic pregnant women as reference.

[b] Model A adjusted for province, year, age, ethnics, IVF, medical insurance, and marital status.

[c] Model B adjusted for all maternal complications during pregnancy in addition to covariates in model A.

[*] p-value < 0.05; [**] p-value < 0.005

© 2022 Shin H et al. *JAMA Network Open*.

eTable 8. Adjusted ORs for maternal and fetal adverse outcomes according to severity of anemia during pregnancy in multiple pregnancies[a]

	Model A [OR (95% CI)][b]					Model B [OR (95% CI)][c]				
	Mild anemia [Hb: 100–109 g/L]	Moderate anemia [Hb: 70–99 g/L]	Severe anemia [Hb: <70 g/L]	Anemia with unknown severity	Overall anemia [Hb: <110 g/L]	Mild anemia [Hb: 100–109 g/L]	Moderate anemia [Hb: 70–99 g/L]	Severe anemia [Hb: <70 g/L]	Anemia with unknown severity	Overall anemia [Hb: <110 g/L]
**Maternal adverse outcomes**										
Placental abruption	1.27 (1.19, 1.35)**	1.61 (1.49, 1.75)**	2.70 (2.18, 3.35)**	1.14 (1.05, 1.23)**	1.32 (1.26, 1.38)**	1.23 (1.16, 1.31)**	1.53 (1.41, 1.66)**	2.26 (1.82, 2.82)**	1.12 (1.03, 1.21)*	1.28 (1.22, 1.34)**
Preterm birth	1.15 (1.13, 1.17)**	1.30 (1.27, 1.33)**	1.68 (1.54, 1.83)**	1.21 (1.19, 1.24)**	1.20 (1.19, 1.22)**	1.11 (1.09, 1.13)**	1.20 (1.17, 1.23)**	1.39 (1.27, 1.51)**	1.16 (1.13, 1.18)**	1.15 (1.13, 1.16)**
Severe postpartum hemorrhage	1.52 (1.45, 1.59)**	3.35 (3.19, 3.51)**	13.07 (11.81, 14.47)**	2.29 (2.19, 2.40)**	2.24 (2.17, 2.31)**	1.38 (1.31, 1.44)**	2.78 (2.64, 2.93)**	10.35 (9.27, 11.56)**	1.93 (1.83, 2.03)**	1.94 (1.87, 2.00)**
Shock	0.80 (0.65, 0.98)*	1.82 (1.48, 2.25)**	26.32 (21.26, 32.58)**	2.31 (1.96, 2.72)**	1.82 (1.62, 2.04)**	0.66 (0.54, 0.82)**	1.20 (0.97, 1.49)	14.31 (11.34, 18.07)**	1.60 (1.33, 1.91)**	1.31 (1.16, 1.48)**
Admission into ICU	0.84 (0.70, 1.01)	1.43 (1.16, 1.75)**	6.39 (4.49, 9.10)**	1.49 (1.28, 1.73)**	1.27 (1.14, 1.42)**	0.69 (0.57, 0.83)**	0.89 (0.72, 1.10)	2.81 (1.93, 4.08)**	1.00 (0.85, 1.18)	0.89 (0.79, 1.00)
Maternal death	0.13 (0.02, 0.92)*	..	..	1.02 (0.40, 2.60)	0.37 (0.16, 0.88)*	0.11 (0.02, 0.81)*	..	..	0.65 (0.23, 1.86)	0.26 (0.10, 0.64)**
Cesarean delivery	1.39 (1.36, 1.41)**	1.56 (1.51, 1.60)**	1.35 (1.23, 1.49)**	1.40 (1.37, 1.43)**	1.42 (1.40, 1.44)**	1.31 (1.28, 1.33)**	1.44 (1.40, 1.49)**	1.14 (1.03, 1.27)**	1.31 (1.27, 1.34)*	1.33 (1.31, 1.35)**
**Fetal adverse outcomes**										
Fetal growth restriction	1.01 (0.97, 1.05)	1.10 (1.04, 1.16)**	1.00 (0.83, 1.21)	1.15 (1.10, 1.20)**	1.07 (1.04, 1.10)**	0.97 (0.94, 1.01)	1.02 (0.97, 1.08)	0.85 (0.71, 1.03)	1.08 (1.03, 1.13)**	1.01 (0.98, 1.04)
Malformation	0.98 (0.94, 1.02)	1.05 (0.99, 1.11)	0.88 (0.71, 1.10)	1.00 (0.95, 1.05)	1.00 (0.97, 1.03)	0.96 (0.92, 1.00)*	1.02 (0.96, 1.08)	0.85 (0.69, 1.06)	0.98 (0.93, 1.03)	0.98 (0.94, 1.01)
Stillbirth	0.75 (0.71, 0.79)**	0.80 (0.74, 0.86)**	1.51 (1.25, 1.82)**	0.67 (0.63, 0.71)**	0.74 (0.72, 0.77)**	0.73 (0.70, 0.77)**	0.78 (0.72, 0.84)**	1.43 (1.18, 1.72)**	0.69 (0.64, 0.73)**	0.74 (0.71, 0.77)**

[a] Non-anemic pregnant women as reference.

[b] Model A adjusted for province, year, age, ethnics, IVF, medical insurance, and marital status.

[c] Model B adjusted for all maternal complications during pregnancy in addition to covariates in model A.

* p-value < 0.05; ** p-value < 0.005.

© 2022 Shin H et al. *JAMA Network Open*.
